# The genome sequence of a soldier beetle, *Cantharis rufa *(Linnaeus, 1758)

**DOI:** 10.12688/wellcomeopenres.19986.1

**Published:** 2023-10-18

**Authors:** Olga Sivell, Duncan Sivell, Michael Geiser, Natural History Museum Genome Acquisition Lab, Darwin Tree of Life Barcoding collective, Wellcome Sanger Institute Tree of Life programme, Wellcome Sanger Institute Scientific Operations: DNA Pipelines collective, Tree of Life Core Informatics collective, Darwin Tree of Life Consortium

**Affiliations:** 1 Natural History Museum, London, England, UK

**Keywords:** Cantharis rufa, soldier beetle, genome sequence, chromosomal, Coleoptera

## Abstract

We present a genome assembly from an individual male *Cantharis rufa* (soldier beetle; Arthropoda; Insecta; Coleoptera; Cantharidae). The genome sequence is 355.3 megabases in span. Most of the assembly is scaffolded into 7 chromosomal pseudomolecules, including the X sex chromosome. The mitochondrial genome has also been assembled and is 17.34 kilobases in length.

## Species taxonomy

Eukaryota; Metazoa; Eumetazoa; Bilateria; Protostomia; Ecdysozoa; Panarthropoda; Arthropoda; Mandibulata; Pancrustacea; Hexapoda; Insecta; Dicondylia; Pterygota; Neoptera; Endopterygota; Coleoptera; Polyphaga; Elateriformia; Elateroidea; Cantharidae; Cantharinae; Cantharis (Linnaeus, 1758) (NCBI:txid350087).

## Background

**Figure 1.**
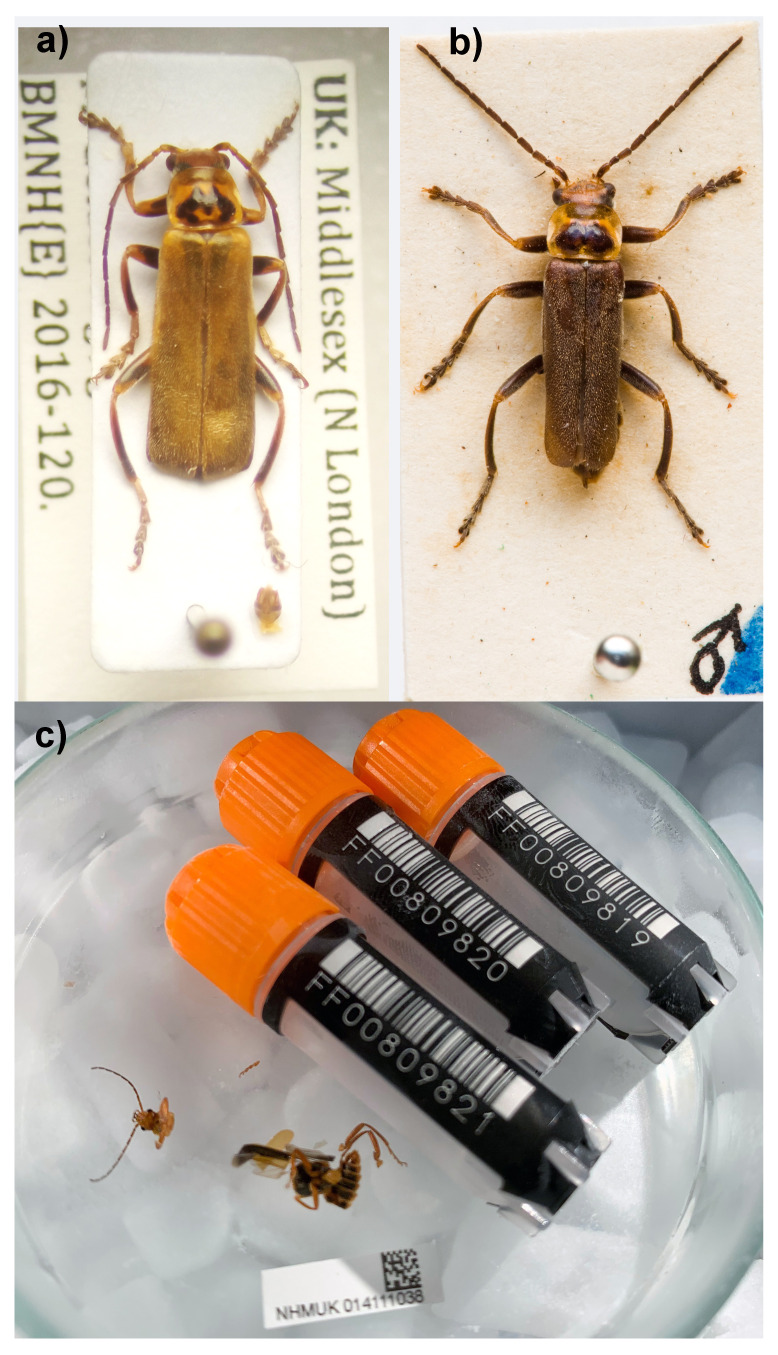
Photographs of *Catharis* rufa specimens. **a**) *Cantharis rufa* Linnaeus, 1758 male from Alexandra Park, N London (Middlesex), 5.vi.2016. Individual representing the colour form with M-shaped dark mark on pronotum (“var. liturata”) commonly found in England and Wales. Specimen number NHMUK010844292. **b**) *Cantharis rufa* Linnaeus, 1758 from Aberlady, E Lothian, Scotland. Male syntype of *Cantharis darwiniana* (Sharp, 1867), a dark colour form unique to Scotland, now treated as a synonym of *C. rufa*. Photo by Derek Croucher. **c**) The *Cantharis rufa* (NHMUK014111038 icCanRufa1) specimen used for genome sequencing.

*Cantharis rufa* Linnaeus, 1758 is a species of beetle from the family Cantharidae (Coleoptera), commonly called soldier beetles. As with other members of the genus the elytra are simple (i.e. without longitudinal or transverse ridges or rows of impressed punctures), weakly sclerotised and soft to the touch ( Figure 1a). The elytra cover the hind wings and abdomen (completely or almost so). The last segment of maxillary palpi is flattened and widened at the apex. The third tarsal segment of the mid and hind legs is bilobed and on the front leg, the front claw has a large basal tooth, while the other claw is simple (all claws are split in the related genus *Rhagonycha*) ( Duff, 2020; Fitton, 1973; Gurney, 2017).

*Cantharis rufa* is 8–11 mm in length and can be easily confused with *Cantharis figurata* Mannerheim, 1843, which is a smaller species (6.5–8 mm). They are both very variable in colour and pattern, each species has dark and pale forms, and a similar range of markings may be present on the head and pronotum ( Gurney, 2017). Within Britain and Europe in general, the darker colour forms are more common towards the north. A form with dark brown elytra and extensive pronotal markings from Scotland was previously treated as a separate species, *C. darwiniana* (Sharp, 1867) ( Figure 1b). The antennae of male *C. figurata* possess large sensory openings on the outer side of segments 4–10, while the antennae of male *C. rufa* lack these; in female *C. rufa* the last visible sternite has a central apical projection, which is not present in *C. figurata* ( Duff, 2020; Fitton, 1973). The male genitalia of the two species also show clear differences ( Dahlgren, 1979): in *C. rufa*, the parameres are long and straight, surpassing the dorsal sclerite of the aedeagus. In *C. figurata*, they are shorter and hidden within the outline of the dorsal sclerite when seen from above.

*Cantharis rufa* is a widely distributed Palaearctic species. It is widespread in Britain, common in England and Wales but scattered in Scotland ( Alexander, 2003; Alexander, 2014; Gurney, 2017). It can be found primarily in open lowland habitats, marshy sites, saltmarshes, deciduous woodlands, parks and hedgerows ( Alexander, 1991; Alexander, 2003). It has been introduced from Europe to the Nearctic where it occurs in the USA and Canada, from Newfoundland west to Ontario south to Massachusetts and New York and the northern Appalachians and has been expanding its range, e.g. to Manitoba ( Pelletier & Hébert, 2014; Semmler & Wrigley, 2015).

The introduced Nearctic populations were formerly assumed to be a separate species, *C. andersoni* Frost, 1922. A peculiar colour form with striped elytra occurs in Central Asia, Siberia and China ( Švihla, 1992). It was originally treated as a separate species, *Cantharis tenuilimbata* (Ballion, 1871), then as a subspecies of *C. rufa* ( Švihla, 1992) and most recently as a synonym ( Kazantsev, 2011). However, the Korean populations previously assigned to *C. rufa* ssp. *tenuilimbata* turned out to belong to a separate species, *C. soeulensis* Pic, 1922. *C. rufa* is not currently recorded from the Korean peninsula ( Kang, 2012).

The species has an annual life cycle with two “prolarval” ("Vorlarvenstadien") and six larval instars, which have been described by Janssen (1963). *Cantharis* larvae are covered with fine straight setae of varying lengths, set at right angles to body surface, giving them an unmistakeable silky appearance. The abdominal segments 1–9 possess pairs of dorsal glandular openings. The mandibular retinaculum is well developed, without an additional tooth, and with a fringe of setae beneath it ( Fitton, 1976). The larvae are predators of small soil and litter-dwelling animals although they can also feed on plant matter and were successfully reared on soaked wheat grains and other plant materials in laboratory conditions ( Janssen, 1963; Payne, 1916). The larvae can be found in soil, under logs, on pathways and even on snow – hence they are often called “snow-worms” ( Luff, 1991). The adults are omnivores: they predate on small invertebrates and also feed on plants. The adults can be found from mid-May to mid-July on herbaceous vegetation, flower heads, trees and shrubs ( Alexander, 1991; Alexander, 2003).

The karyotype of *Cantharis rufa* was described and illustrated by James and Angus (2007). Here we present a chromosomally complete genome sequence for *Cantharis rufa*, based on a male specimen from Luton. It will aid research on the taxonomy, phylogeny and ecology of this species and the family. The high-quality genome of *Cantharis rufa* was sequenced as part of the Darwin Tree of Life Project, a collaborative effort to sequence all named eukaryotic species in the Atlantic Archipelago of Britain and Ireland.

## Genome sequence report

The genome was sequenced from one male *Cantharis rufa* ( Figure 1c) collected from Wigmore Park, Luton, England (51.88, –0.37). A total of 67-fold coverage in Pacific Biosciences single-molecule HiFi long reads was generated. Primary assembly contigs were scaffolded with chromosome conformation Hi-C data. Manual assembly curation corrected 38 missing joins or mis-joins and removed 9 haplotypic duplications, reducing the assembly length by 1.39% and the scaffold number by 24%, and increasing the scaffold N50 by 23.14%.

**Figure 2.**
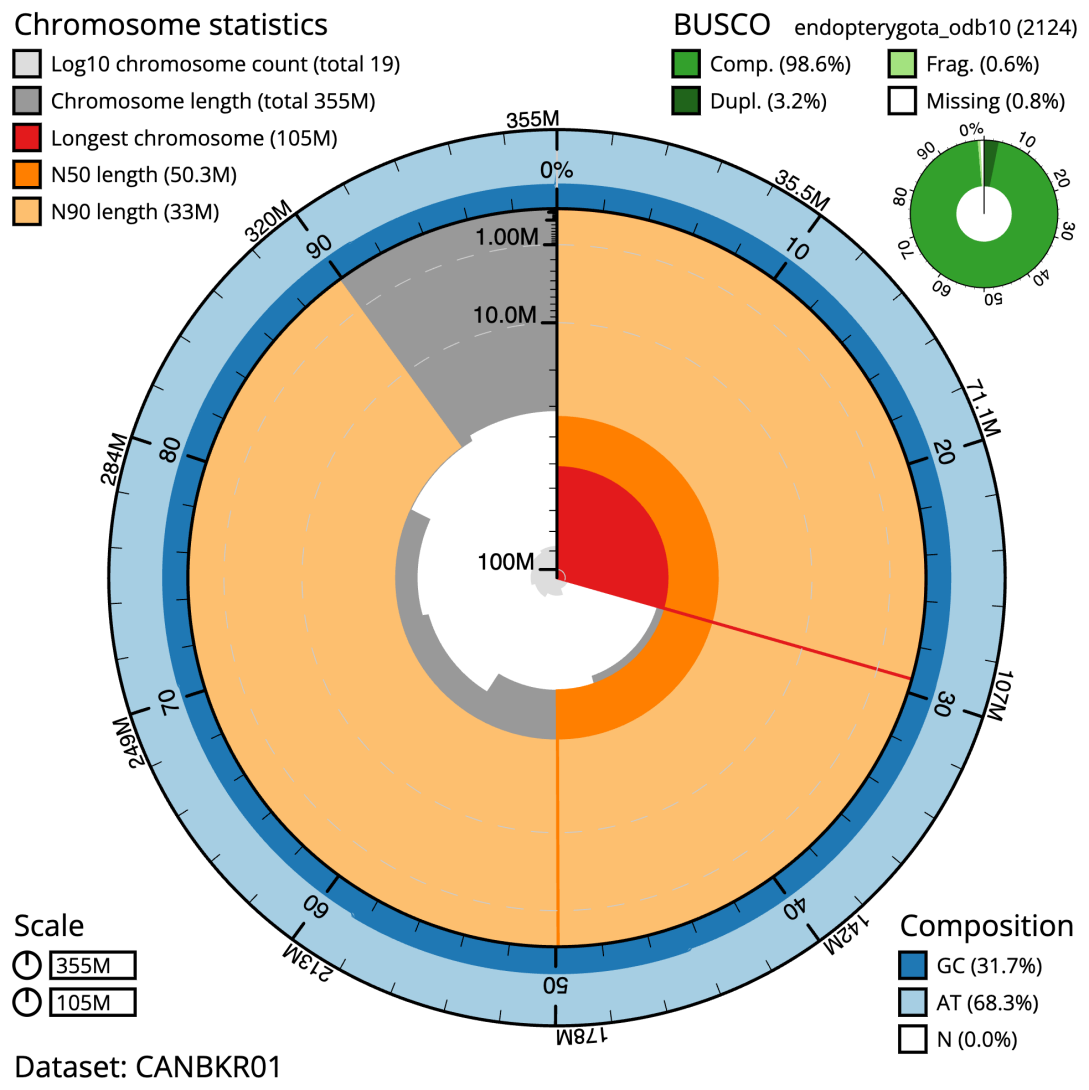
Genome assembly of *Cantharis rufa*, icCanRufa1.1: metrics. The BlobToolKit Snailplot shows N50 metrics and BUSCO gene completeness. The main plot is divided into 1,000 size-ordered bins around the circumference with each bin representing 0.1% of the 355,331,611 bp assembly. The distribution of scaffold lengths is shown in dark grey with the plot radius scaled to the longest scaffold present in the assembly (104,616,211 bp, shown in red). Orange and pale-orange arcs show the N50 and N90 scaffold lengths (50,306,085 and 32,996,793 bp), respectively. The pale grey spiral shows the cumulative scaffold count on a log scale with white scale lines showing successive orders of magnitude. The blue and pale-blue area around the outside of the plot shows the distribution of GC, AT and N percentages in the same bins as the inner plot. A summary of complete, fragmented, duplicated and missing BUSCO genes in the endopterygota_odb10 set is shown in the top right. An interactive version of this figure is available at https://blobtoolkit.genomehubs.org/view/icCanRufa1.1/dataset/CANBKR01/snail.

The final assembly has a total length of 355.3 Mb in 18 sequence scaffolds with a scaffold N50 of 50.3 Mb ( Table 1). Most (99.9%) of the assembly sequence was assigned to 7 chromosomal-level scaffolds, representing 6 autosomes and the X sex chromosome. Chromosome-scale scaffolds confirmed by the Hi-C data are named in order of size ( Figure 2– Figure 5; Table 2). While not fully phased, the assembly deposited is of one haplotype. Contigs corresponding to the second haplotype have also been deposited. The mitochondrial genome was also assembled and can be found as a contig within the multifasta file of the genome submission.

**Table 1.** Genome data for *Cantharis rufa*, icCanRufa1.1.

Project accession data		
Assembly identifier	icCanRufa1.1	
Species	*Cantharis rufa*	
Specimen	icCanRufa1	
NCBI taxonomy ID	350087	
BioProject	PRJEB56058	
BioSample ID	SAMEA7520947	
Isolate information	icCanRufa1, male: thorax (DNA sequencing and Hi-C scaffolding)	
Assembly metrics *		*Benchmark*
Consensus quality (QV)	64.8	*≥ 50*
*k*-mer completeness	100%	*≥ 95%*
BUSCO **	C:98.6%[S:95.4%,D:3.2%],F:0.6%,M: 0.8%,n:2,124	*C ≥ 95%*
Percentage of assembly mapped to chromosomes	99.9%	*≥ 95%*
Sex chromosomes	X chromosome	*localised homologous pairs*
Organelles	Mitochondrial genome assembled	*complete single alleles*
Raw data accessions		
PacificBiosciences SEQUEL II	ERR10224927, ERR10224928	
Hi-C Illumina	ERR10297822	
Genome assembly		
Assembly accession	GCA_947369205.1	
*Accession of alternate* *haplotype*	GCA_947369215.1	
Span (Mb)	355.3	
Number of contigs	86	
Contig N50 length (Mb)	10.5	
Number of scaffolds	18	
Scaffold N50 length (Mb)	50.3	
Longest scaffold (Mb)	104.6	

* Assembly metric benchmarks are adapted from column VGP-2020 of “Table 1: Proposed standards and metrics for defining genome assembly quality” from ( Rhie *et al.*, 2021). ** BUSCO scores based on the endopterygota_odb10 BUSCO set using v5.3.2. C = complete [S = single copy, D = duplicated], F = fragmented, M = missing, n = number of orthologues in comparison. A full set of BUSCO scores is available at https://blobtoolkit.genomehubs.org/view/icCanRufa1.1/dataset/CANBKR01/busco.

**Figure 3.**
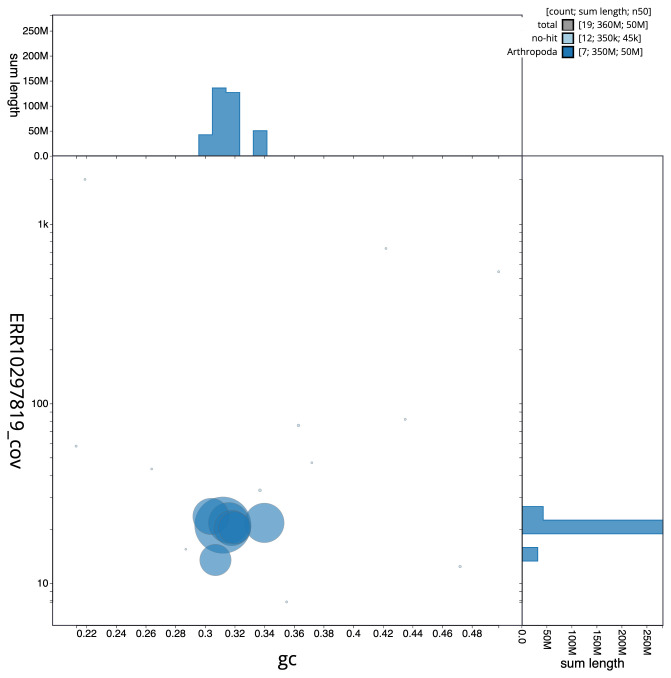
Genome assembly of *Cantharis rufa*, icCanRufa1.1: BlobToolKit GC-coverage plot. Scaffolds are coloured by phylum. Circles are sized in proportion to scaffold length. Histograms show the distribution of scaffold length sum along each axis. An interactive version of this figure is available at https://blobtoolkit.genomehubs.org/view/icCanRufa1.1/dataset/CANBKR01/blob.

**Figure 4.**
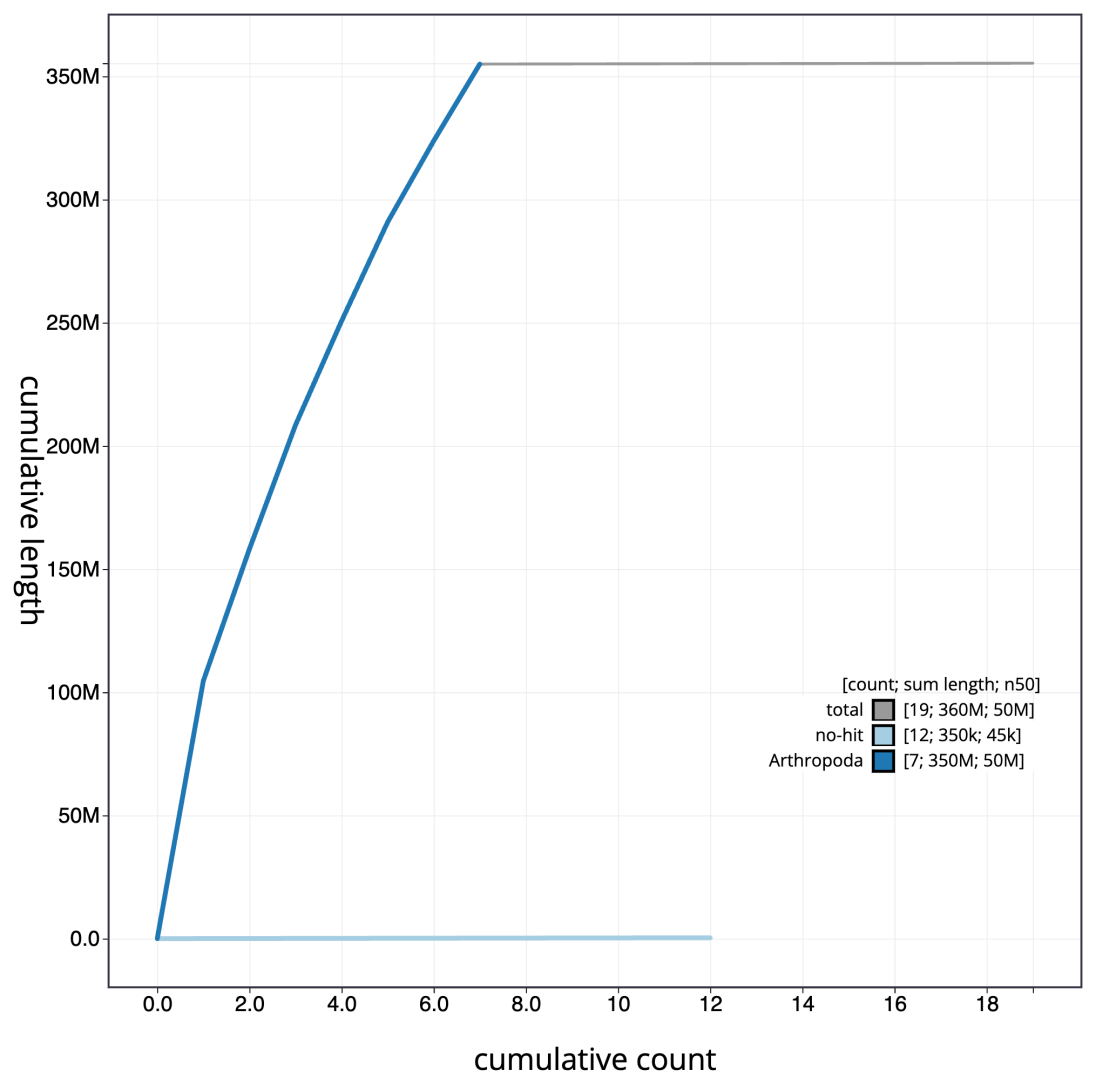
Genome assembly of *Cantharis rufa*, icCanRufa1.1: BlobToolKit cumulative sequence plot. The grey line shows cumulative length for all scaffolds. Coloured lines show cumulative lengths of scaffolds assigned to each phylum using the buscogenes taxrule. An interactive version of this figure is available at https://blobtoolkit.genomehubs.org/view/icCanRufa1.1/dataset/CANBKR01/cumulative.

**Figure 5.**
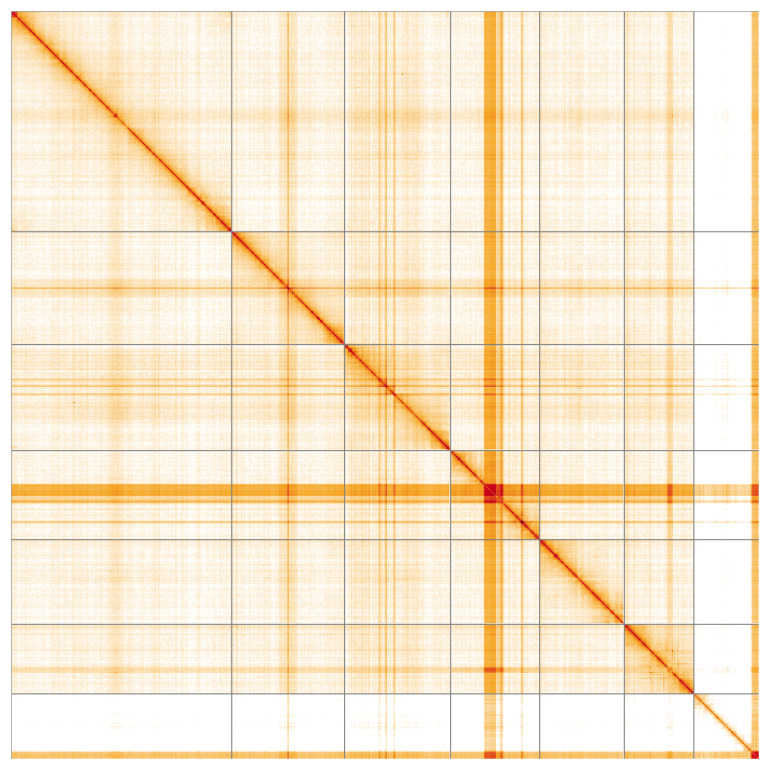
Genome assembly of *Cantharis rufa*, icCanRufa1.1: Hi-C contact map of the icCanRufa1.1 assembly, visualised using HiGlass. Chromosomes are shown in order of size from left to right and top to bottom. An interactive version of this figure may be viewed at https://genome-note-higlass.tol.sanger.ac.uk/l/?d=UwGVGhAYToicL07o3d5k0g.

**Table 2.** Chromosomal pseudomolecules in the genome assembly of *Cantharis rufa*, icCanRufa1.

INSDC accession	Chromosome	Length (Mb)	GC%
OX376303.1	1	104.62	31.0
OX376304.1	2	53.52	31.5
OX376305.1	3	50.31	34.0
OX376306.1	4	42.26	30.5
OX376307.1	5	40.15	32.0
OX376308.1	6	33.0	32.0
OX376309.1	X	31.15	30.5
OX376310.1	MT	0.02	22.0

The estimated Quality Value (QV) of the final assembly is 64.8 with *k*-mer completeness of 100%, and the assembly has a BUSCO v5.3.2 completeness of 98.6% (single = 95.4%, duplicated = 3.2%), using the endopterygota_odb10 reference set ( *n* = 2,124).

Metadata for specimens, spectral estimates, sequencing runs, contaminants and pre-curation assembly statistics can be found at https://links.tol.sanger.ac.uk/species/350087.

## Methods

### Sample acquisition and nucleic acid extraction

One male specimen (NHMUK014111038, icCanRufa1) of *Cantharis rufa* ( Figure 1) was hand-picked from Wigmore Park, Luton, England (51.88, –0.36) on 2020-06-02 by Olga Sivell. It was identified by Duncan Sivell, Natural History Museum, London following Fitton (1973). The specimen was snap-frozen using dry ice and the tissue samples taken from it were stored in a CoolRack prior to genome sequencing.

DNA was extracted at the Tree of Life laboratory, Wellcome Sanger Institute (WSI). The icCanRufa1 sample was weighed and dissected on dry ice with tissue set aside for Hi-C sequencing. Tissue from the thorax was disrupted using a Nippi Powermasher fitted with a BioMasher pestle. High molecular weight (HMW) DNA was extracted using the Qiagen MagAttract HMW DNA extraction kit. HMW DNA was sheared into an average fragment size of 12–20 kb in a Megaruptor 3 system with speed setting 30. Sheared DNA was purified by solid-phase reversible immobilisation using AMPure PB beads with a 1.8X ratio of beads to sample to remove the shorter fragments and concentrate the DNA sample. The concentration of the sheared and purified DNA was assessed using a Nanodrop spectrophotometer and Qubit Fluorometer and Qubit dsDNA High Sensitivity Assay kit. Fragment size distribution was evaluated by running the sample on the FemtoPulse system.

### Sequencing

Pacific Biosciences HiFi circular consensus DNA sequencing libraries were constructed according to the manufacturers’ instructions. DNA sequencing was performed by the Scientific Operations core at the WSI on Pacific Biosciences SEQUEL II (HiFi) and HiSeq X Ten (10X) instruments. Hi-C data were also generated from remaining thorax tissue of icCanRufa1 using the Arima2 kit and sequenced on the Illumina NovaSeq 6000 instrument.

### Genome assembly, curation and evaluation

Assembly was carried out with Hifiasm ( Cheng *et al.*, 2021) and haplotypic duplication was identified and removed with purge_dups ( Guan *et al.*, 2020). The assembly was then scaffolded with Hi-C data ( Rao *et al.*, 2014) using YaHS ( Zhou *et al.*, 2023). The assembly was checked for contamination and corrected as described previously ( Howe *et al.*, 2021). Manual curation was performed using HiGlass ( Kerpedjiev *et al.*, 2018) and Pretext ( Harry, 2022). The mitochondrial genome was assembled using MitoHiFi ( Uliano-Silva *et al.*, 2023), which runs MitoFinder ( Allio *et al.*, 2020) or MITOS ( Bernt *et al.*, 2013) and uses these annotations to select the final mitochondrial contig and to ensure the general quality of the sequence.

A Hi-C map for the final assembly was produced using bwa-mem2 ( Vasimuddin *et al.*, 2019) in the Cooler file format ( Abdennur & Mirny, 2020). To assess the assembly metrics, the *k*-mer completeness and QV consensus quality values were calculated in Merqury ( Rhie *et al.*, 2020). This work was done using Nextflow ( Di Tommaso *et al.*, 2017) DSL2 pipelines “sanger-tol/readmapping” ( Surana *et al.*, 2023a) and “sanger-tol/genomenote” ( Surana *et al.*, 2023b). The genome was analysed within the BlobToolKit environment ( Challis *et al.*, 2020) and BUSCO scores ( Manni *et al.*, 2021; Simão *et al.*, 2015) were calculated.

Table 3 contains a list of relevant software tool versions and sources.

**Table 3.** Software tools: versions and sources.

Software tool	Version	Source
BlobToolKit	4.1.5	https://github.com/blobtoolkit/blobtoolkit
BUSCO	5.3.2	https://gitlab.com/ezlab/busco
Hifiasm	0.16.1-r375	https://github.com/chhylp123/hifiasm
HiGlass	1.11.6	https://github.com/higlass/higlass
Merqury	MerquryFK	https://github.com/thegenemyers/MERQURY.FK
MitoHiFi	2	https://github.com/marcelauliano/MitoHiFi
PretextView	0.2	https://github.com/wtsi-hpag/PretextView
purge_dups	1.2.3	https://github.com/dfguan/purge_dups
sanger-tol/genomenote	v1.0	https://github.com/sanger-tol/genomenote
sanger-tol/readmapping	1.1.0	https://github.com/sanger-tol/readmapping/tree/1.1.0
YaHS	yahs-1.1.91eebc2	https://github.com/c-zhou/yahs

### Wellcome Sanger Institute – Legal and Governance

The materials that have contributed to this genome note have been supplied by a Darwin Tree of Life Partner. The submission of materials by a Darwin Tree of Life Partner is subject to the **‘Darwin Tree of Life Project Sampling Code of Practice’**, which can be found in full on the Darwin Tree of Life website here. By agreeing with and signing up to the Sampling Code of Practice, the Darwin Tree of Life Partner agrees they will meet the legal and ethical requirements and standards set out within this document in respect of all samples acquired for, and supplied to, the Darwin Tree of Life Project. 

Further, the Wellcome Sanger Institute employs a process whereby due diligence is carried out proportionate to the nature of the materials themselves, and the circumstances under which they have been/are to be collected and provided for use. The purpose of this is to address and mitigate any potential legal and/or ethical implications of receipt and use of the materials as part of the research project, and to ensure that in doing so we align with best practice wherever possible. The overarching areas of consideration are:

• Ethical review of provenance and sourcing of the material

• Legality of collection, transfer and use (national and international) 

Each transfer of samples is further undertaken according to a Research Collaboration Agreement or Material Transfer Agreement entered into by the Darwin Tree of Life Partner, Genome Research Limited (operating as the Wellcome Sanger Institute), and in some circumstances other Darwin Tree of Life collaborators.

## Data Availability

European Nucleotide Archive: *Cantharis rufa*. Accession number PRJEB56058; https://identifiers.org/ena.embl/PRJEB56058. ( Wellcome Sanger Institute, 2022) The genome sequence is released openly for reuse. The *Cantharis rufa* genome sequencing initiative is part of the Darwin Tree of Life (DToL) project. All raw sequence data and the assembly have been deposited in INSDC databases. The genome will be annotated using available RNA-Seq data and presented through the Ensembl pipeline at the European Bioinformatics Institute. Raw data and assembly accession identifiers are reported in Table 1.

## Author information

Members of the Natural History Museum Genome Acquisition Lab are listed here: https://doi.org/10.5281/zenodo.4790042.

Members of the Darwin Tree of Life Barcoding collective are listed here: https://doi.org/10.5281/zenodo.4893703.

Members of the Wellcome Sanger Institute Tree of Life programme are listed here: https://doi.org/10.5281/zenodo.4783585.

Members of Wellcome Sanger Institute Scientific Operations: DNA Pipelines collective are listed here: https://doi.org/10.5281/zenodo.4790455.

Members of the Tree of Life Core Informatics collective are listed here: https://doi.org/10.5281/zenodo.5013541.

Members of the Darwin Tree of Life Consortium are listed here: https://doi.org/10.5281/zenodo.4783558.
