# Supplementary material for: The genome sequence of a soldier beetle, Cantharis rufa (Linnaeus, 1758)
Source: Wellcome Open Res. 2023 Oct 18;8:478. [Version 1] doi: 10.12688/wellcomeopenres.19986.1 (PMC10973763; doi:10.12688/wellcomeopenres.19986.1)
Supplement: Peer review history

# Reviewer report: Reviewer response for version 1

**DOI:** 10.21956/wellcomeopenres.22132.r76093

**Published:** 2024-03-27

**Authors:** Michael G. Ritchie

**Affiliations:** 1 Centre for Biological Diversity, University of St Andrews, St Andrews, Scotland, UK

This is a high quality genome of the soldier beetle prepared to an excellent standard. It will be very useful for comparative and other work.

 I have only two very minor queries which are suggestions rather than essential revisions. 

 Why was soeulensis judged to be a separate species, what led to this conclusion?

 Given that there are two 'easily confused' species, what features were decisive in identifying this one as rufa? Is there a photo of e.g. genitalia providing definitive evidence?

Are sufficient details of methods and materials provided to allow replication by others?

Yes

Is the rationale for creating the dataset(s) clearly described?

Yes

Are the datasets clearly presented in a useable and accessible format?

Yes

Are the protocols appropriate and is the work technically sound?

Yes

Reviewer Expertise:

Evolutionary biology, genetics

I confirm that I have read this submission and believe that I have an appropriate level of expertise to confirm that it is of an acceptable scientific standard.

# Reviewer report: Reviewer response for version 1

**DOI:** 10.21956/wellcomeopenres.22132.r72874

**Published:** 2024-02-13

**Authors:** Danilo Amaral

**Affiliations:** 1 Universidade Federal do ABC (UFABC), São Paulo, Brazil

Dear all,

 I am pleased for the opportunity to review this manuscript. The manuscript demonstrates an engaging and accessible approach, offering readers a detailed understanding of the morphological characteristics, geographical distribution, and genomic aspects of this beetle species. The narrative is clear and well-structured, making the complexity of taxonomy and genomics accessible to a diverse audience. The quality of the work is noteworthy, particularly in the detailed presentation of the complete genomic sequence, encompassing everything from sample collection to assembly metric analysis. The inclusion of data on geographical distribution, life cycle, and distinctive features in comparison to similar species enriches the overall understanding of Cantharis rufa. However, there are some suggestions that could further enhance the impact of the article. I recommend that the authors include a conclusion paragraph to highlight the most significant findings and the potential impact of these results on taxonomic and ecological research. Additionally, they may consider expanding the explanation of how the genomic sequence can be applied in future studies, emphasizing potential implications for understanding the species' evolution. These suggestions aim to further strengthen the quality of the work, providing an even more enriching reading experience and ensuring a comprehensive understanding of the presented findings.

Are sufficient details of methods and materials provided to allow replication by others?

Yes

Is the rationale for creating the dataset(s) clearly described?

Yes

Are the datasets clearly presented in a useable and accessible format?

Yes

Are the protocols appropriate and is the work technically sound?

Yes

Reviewer Expertise:

Bioinformatics

I confirm that I have read this submission and believe that I have an appropriate level of expertise to confirm that it is of an acceptable scientific standard.
